# Short-Term Safety and Efficacy of PreserFlo™ Microshunt in Patients with Refractory Intraocular Pressure Elevation After Dexamethasone Implant Intravitreal Injection

**DOI:** 10.3390/jcm14020507

**Published:** 2025-01-14

**Authors:** Leonie Bourauel, Michael Petrak, Frank G. Holz, Karl Mercieca, Constance Weber

**Affiliations:** Department of Ophthalmology, University of Bonn, 53127 Bonn, Germany

**Keywords:** PreserFlo MicroShunt, glaucoma, glaucoma surgery, dexamethasone, IOP

## Abstract

**Background**: The PreserFlo™ MicroShunt (PFMS) is a bleb-forming device considered to be less invasive than traditional glaucoma surgery such as trabeculectomy. This study evaluates the 1-year success rates as well as safety profile of PFMS in patients having high intraocular pressure (IOP) and/or glaucoma refractory to drop therapy with a history of prior intravitreal dexamethasone therapy. **Methods**: A total of 16 eyes after PFMS implantation due to elevated IOP after intravitreal dexamethasone implant (DEX-I) administration were included in this retrospective cohort study. Success rates and secondary outcomes were evaluated. **Results**: Qualified and complete success rates at 12 months, respectively, were 14/16 and 12/16 eyes for criterion A, 13/16 and 11/16 eyes for B, 13/16 and 11/16 eyes for C, and 6/16 and 6/16 eyes for D. The overall mean (range) preoperative IOP decreased from 27 (16–38) mmHg to 13 (10–17) mmHg at 12 months. BCVA was not significantly different up to 12 months (*p* = 0.63). The preoperative mean (range) number of medications decreased from 3.56 (2–4) to 0.31 (0–3) at 12 months. One eye underwent needling twice, and two eyes were revised surgically. One patient needed replacement of the PFMS. There were no hypotony-related complications. **Conclusions**: The PFMS is an effective surgical option for patients with steroid-induced IOP elevation. It demonstrates satisfactory short-term success rates, a reduced need for pressure-lowering eye drops, an excellent safety profile with minimal postoperative care, and a low complication rate. Additional interventions such as needling or revisions were infrequently necessary. However, PFMS may not be the ideal choice for cases requiring a low target pressure (≤12 mmHg).

## 1. Introduction

Glaucoma is a major cause of irreversible visual impairment worldwide and leads to reduced quality of life due to impaired visual health [1,2]. Therapeutic measures depend on the severity of the disease. If intraocular pressure (IOP)-lowering eye drops or laser treatment do not lead to a sufficient reduction in IOP [3,4], surgical measures are recommended. As an alternative to conventional glaucoma surgeries, ‘bleb-forming devices’ were developed especially for patients with early forms of glaucoma as well as to reduce postoperative complications and medication burden [5]. Devices like the PreserFlo™ MicroShunt (PFMS, Santen, Miami, FL, USA) are less invasive than traditional bleb-forming surgeries. With an internal lumen of 70 µm, the PFMS creates a posterior filtering bleb and facilitates controlled fluid flow while significantly reducing the risk of postoperative hypotony [6]. This approach reduces the need for postoperative follow-up visits and interventions, which likely contributed to the increased use of PFMS implants during the COVID-19 pandemic [7]. The relatively straightforward learning curve, moderate IOP reduction, and favorable safety profile have made the PFMS an increasingly popular treatment option for patients with progressing glaucoma while on IOP-lowering drops, as long as a particularly low IOP is not required [8,9,10].

Intravitreal administration of anti-VEGF antibodies or corticosteroids is standard for the treatment of macular edema (ME) of various causes. The sustained-release intravitreal dexamethasone 0.7 mg implant (Ozurdex^®^, AbbVie Inc., North Chicago, IL, USA; hereafter abbreviated as DEX-I) is usually used as a second-line therapy and has an effective duration of up to six months [11,12]. Dexamethasone inhibits inflammatory cytokines, and therefore suppresses inflammation [13]. However, a major complication is a potential rise in IOP, with peak pressure at two months after injection [14,15]. An IOP increase after DEX-I has been detected in up to 27% percent of patients, with IOP-lowering medication sufficient to control this in most cases [16]. If drops do not lead to adequate IOP reduction, filtering surgeries or laser therapy can be carried out [16,17]. Especially in cases of elevated IOP without manifest glaucomatous damage, the chosen surgical approach should prioritize minimizing complications and postoperative risks while still ensuring effective IOP reduction to prevent potential damage. However, there is currently no consensus on the most appropriate surgical procedure for this particular patient cohort, as surgery is rarely required. Consequently, the reported case numbers for various procedures remain low [17,18,19,20], and to date, only a single case report has documented PFMS implantation for refractory ocular hypertension following intravitreal DEX-I administration [21].

Given its favorable attributes, PFMS presents itself as a promising option for achieving low-risk and effective IOP reduction. Nonetheless, the literature on PFMS use in specific glaucoma subtypes remains sparse, underscoring the need for further investigation. Two- and three-year results have shown that PFMS is generally considered safe and effective in terms of its IOP-lowering effect [22,23]. Detailed data on surgical procedures are crucial for assessing their efficacy, enabling a clear understanding of their benefits and limitations. This knowledge is vital for identifying the patients who are most likely to benefit from a given procedure. The aim of this study was to determine the efficacy and safety of PFMS implantation in patients who developed a steroid response following repeated intravitreal DEX-I therapy.

## 2. Materials and Methods

### 2.1. Patients

The study protocol conformed to the ethical guidelines of the 2000 Declaration of Helsinki as reflected in a priori approval by the institution’s Human Research Committee.

Medical records of patients who underwent PFMS surgery with Mitomycin C (MMC) augmentation at the University Eye Hospital Bonn from April 2021 to October 2023 were reviewed, and patients aged 18 years or older who had PFMS surgery due to steroid-induced IOP rise after DEX-I administration were included. Patients with steroid-induced ocular hypertension (SIOH) or steroid-induced glaucoma (SIG) and complete follow-up data after a minimum of 12 months were included in the study. Patients already known to have glaucoma prior to DEX-I therapy, that was previously well controlled under local therapy, were included. Two experienced glaucoma surgeons performed surgeries according to local protocol. Table 1 shows the underlying ocular conditions requiring DEX-I administration. We included the patient’s data at the time of surgery, and follow-up data were retrospectively collected at each visit. All patients had a comprehensive ophthalmic examination, including BCVA (Snellen chart converted to logMAR), IOP (Goldmann applanation tonometry), slit lamp biomicroscopy, and fundus biomicroscopy. Preoperative variables included sex, age, glaucoma type, BCVA, and IOP. Follow-up data included BCVA, IOP, visual fields, complications, postoperative glaucoma medications, interventions, and factors associated with failure. Data were collected at day 1, week 1, month 1, month 3, month 6, and month 12 after surgery. Intervals for each time point are based on recommendations for the design and reporting of glaucoma surgical trials by the World Glaucoma Association [24].

### 2.2. Surgical Technique

All patients gave informed consent prior to surgery. Two experienced surgeons used similar surgical techniques based on the local protocol and as described previously [25]. All procedures were performed under sub-Tenon’s or subconjunctival anesthesia. The cornea was fixed with a traction suture, and a fornix-based conjunctival flap was created. Bleeding vessels were minimally cauterized before corneal sponges soaked with MMC were placed under the sub-Tenon’s pocket. The concentration of MMC was 0.2 mg/mL, and it was applied for two minutes. A 2 mm long scleral tunnel was created 3 mm behind the marked limbus using a 1 mm specialized micro-knife, and the anterior chamber was opened with a 25-gauge needle. The implant was inserted through the tunnel, with the fins positioned inside the sclera. The Tenon’s and conjunctival layers were advanced over and above the tube with 10-0 nylon sutures being used to secure both layers back to their anatomical position. Postoperatively, a standardized regimen of topical antibiotics and steroids was prescribed, which was gradually reduced over two to three months.

### 2.3. Success Criteria

We followed the recommendation of the ‘Guidelines on Design and Report of Glaucoma Surgical Trials’ of the World Glaucoma Association for the definition of success criteria [24]. Four different success criteria were used, based on IOP thresholds and percentage IOP reduction from baseline: (1) Criterion A: IOP ≤ 21 mmHg, reduction ≥ 20%; (2) Criterion B: IOP ≤ 18 mmHg, reduction ≥ 20%; (3) Criterion C: IOP ≤ 15 mmHg, reduction ≥ 30%; (4) Criterion D: IOP ≤ 12 mmHg, reduction ≥ 30%. Success was defined as complete if it was reached without glaucoma medication and as qualified if reached with or without glaucoma medication. Failure was considered when the above-mentioned criteria were not fulfilled at any postoperative visit after three months or if one of the following occurred: loss of light perception; hypotony-related complications; IOP below 6 mmHg or over 21 mmHg; inadequate IOP control requiring acetazolamide, surgical revision, or further glaucoma surgery.

The primary outcome included success rates based on the criteria above. Secondary outcomes were mean IOP, BCVA, number of IOP-lowering drops, and complications. All analyses were conducted on a de-identified dataset. Additionally, we used the same success criteria as in the studies previously conducted by our group and other studies on PFMS in order to facilitate comparison of the outcomes.

### 2.4. Statistical Analysis

Statistical analysis was performed with GraphPad Prism 9.5.1 for Windows (GraphPad Software, Boston, MA, USA). BCVA values were converted to the logMAR scale prior to statistical analysis. First, data distribution of all variables was checked for normality using the Shapiro–Wilk test and visually with frequency histograms. As data were not normally distributed, the Mann–Whitney U test was used for the numerical variables preoperative and postoperative visual acuity (logMAR) as well as pre- and postoperative IOP. We considered *p* values < 0.05 to be statistically significant. Time-dependent survival probabilities were estimated with the Kaplan–Meier method.

## 3. Results

A total of 16 eyes of 15 patients who underwent PFMS surgery were included in this study. All patients were of European descent. Demographics and baseline clinical characteristics are displayed in Table 1.

**Figure 1:**
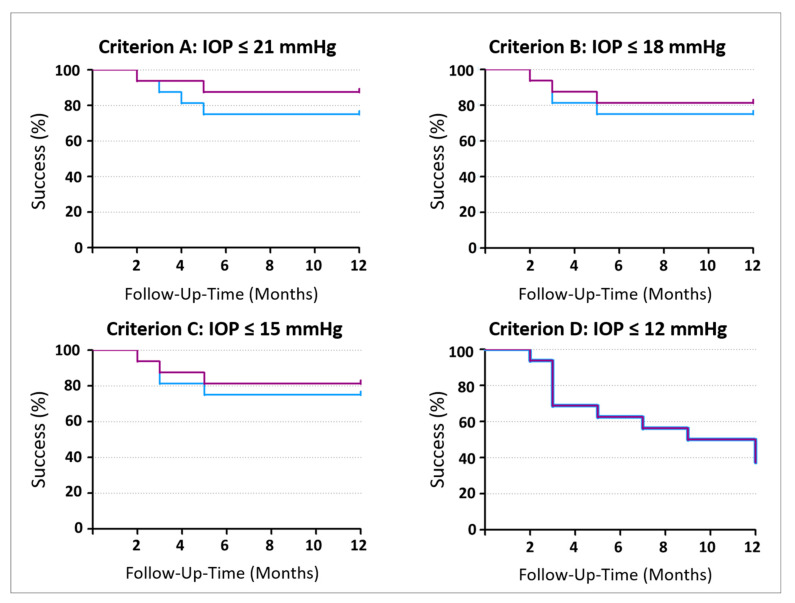
Kaplan–Meier curves depicting qualified (purple) and complete (blue) success rates 12 months after surgery. For Criterion D, qualified and complete success rates were identical. The *x*-axis in Figure 1 incorporates symmetrical monthly ranges because failure was assessed at each monthly interval, even outside the study’s protocol.

Primary outcomes after 12 months (n = 16) were related to the above-mentioned success criteria: for Criterion A, qualified and complete success rates were 14/16 and 12/16 eyes at 12 months. For Criterion B, these were 13/16 eyes and 11/16 eyes; for Criterion C, 13/16 eyes and 11/16 eyes; and for Criterion D, 6/16 eyes and 6/16 eyes at 12 months (Figure 1).

Postoperative complications and further procedures related to PFMS implantation are shown in Table 2. Two eyes (12.5%) failed because of inadequate IOP control. One patient received needling with 5-fluorouracil administration twice; two eyes were revised surgically due to an encapsulated bleb. One patient needed PFMS exchange because the tip of the tube was obstructed by material deposition. Hypotony with choroidal detachment occurred in one patient at week 1 and resolved without further intervention by the one-month post-op visit.

**Figure 2:**
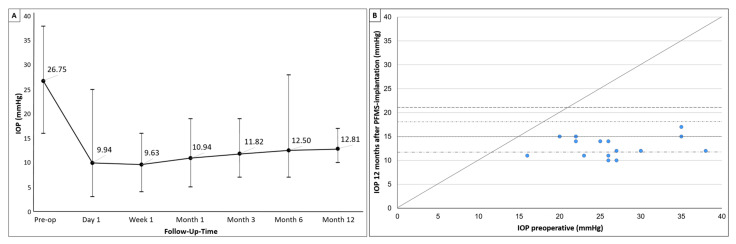
(**A**) IOP development preoperatively and at each follow-up time point: Mean IOP decreased from 27 mmHg (16–38 mmHg) to 13 mmHg (10–17 mmHg) (reduction of 51%) after 12 months. (**B**) Scatterplot of preoperative versus postoperative IOP values at 12 months after surgery. Dots represent eyes, and horizontal lines indicate IOP used as threshold for success criteria.

The mean preoperative IOP (range, median, interquartile range) was 27 mmHg (16–38 mmHg, 26 mmHg, 23–30 mmHg). It decreased to 13 mmHg (7–28 mmHg, 12 mmHg, 10–13 mmHg) after 6 months with a mean reduction of 51.5% (6.7–81.6%, 51.8%, 45.1–61.9%) and 13 mmHg (10–17 mmHg, 12 mmHg, 11–14 mmHg) with a mean reduction of 50.1% (25–68.4%, 53.9%, 42.1–60%) after 12 months (Figure 2). At each postoperative time point, the IOP values were significantly lower than the preoperative values (*p* < 0.0001).

The lowest IOP values were recorded one week after PFMS implantation, followed by a slight increase observed up to the 6-month follow-up visit. The IOP values at day 1 were significantly lower than those at 6 months (*p* = 0.032, *p* = 0.058, *p* = 0.353) and 12 months (*p* = 0.003, *p* = 0.005, *p* = 0.053); there was also a significant difference between the IOP values at week 1 and month 12 (*p* = 0.005). The IOP values at 1, 3, 6, and 12 months did not differ significantly from each other (*p* = 0.053, *p* = 0.159, *p* = 0.246). As shown in Figure 2, the IOP at 12 months after surgery was not equal to or higher than the preoperative IOP in any eye.

**Figure 3:**
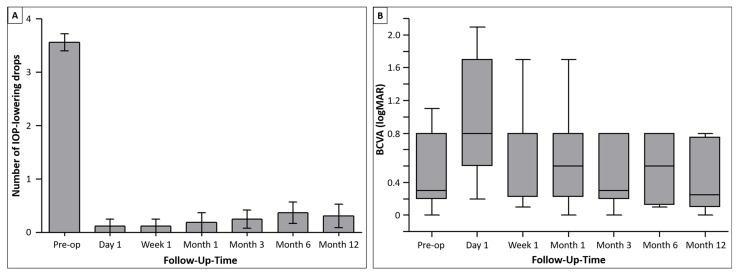
(**A**) Mean amount of pressure-lowering eye drops: the number of IOP-lowering medications decreased from 3.56 agents (2–4) to 0.31 (0–3) after 12 months. (**B**) BCVA development: there was no significant change in BCVA 12 months after surgery compared with preoperative values.

The mean number of topical IOP-lowering agents before surgery was 3.56 (2–4; 4; 3–4). It decreased significantly to 0.38 (0–2; 0; 0–0) after 6 months and 0.31 (0–3; 0; 0–0) at 12 months (Figure 3). At all the time points following surgery, the number of medications was significantly lower compared with preoperative levels (*p* < 0.0001; Figure 3), with 14 out of 16 eyes requiring no IOP-lowering eye drops after 12 months. Continued DEX-I therapy appeared to have no influence on the need for IOP-lowering drops following PFMS surgery.

The mean (range; median; interquartile range) BCVA preoperatively was 0.51 logMAR (0–1.3 logMAR; 0.3 logMAR; 0.2–1.0 logMAR), and it approached preoperative values at 6 months post-surgery with 0.56 logMAR (0.1–1.0 logMAR; 0.6 logMAR; 0.18–1.0 logMAR). After 12 months, BCVA decreased to 0.46 logMAR (0–1.0 logMAR; 0.25 logMAR; 0.1–0.85 logMAR). BCVA was not significantly different at 12 months compared with preoperative values (*p* = 0.63).

Within 12 months after surgery, 12 out of 16 patients were treated with DEX-I for macular edema at least once. Among these, only one required pressure-lowering eye drops. In another case, the PFMS was replaced prior to DEX-I administration, and the IOP remained stable even with subsequent steroid treatment.

## 4. Discussion

PFMS is widely recognized as an effective and safe procedure for glaucoma treatment. However, limited data exist on the specific patient groups that are most likely to benefit from implantation. Notably, only one case report to date has addressed PFMS implantation for steroid response [21]. This is, to the best of our knowledge, the first reported cohort of patients with PFMS implantation due to refractory IOP elevation following intravitreal dexamethasone administration.

There is no standardized definition of a steroid-induced rise in IOP (‘steroid response’). However, a frequently used definition is an IOP increase ≥10 mmHg with clinical relevance [26]. If SIOH is not adequately managed, it can lead to the development of steroid-induced glaucoma. In this study, we report on patients with both SIOH and SIG, as we believe that this broader inclusion best reflects the realities of everyday clinical practice. Additionally, some of our patients had pre-existing open-angle glaucoma prior to initiating DEX-I therapy. However, before steroid treatment, their IOP was well controlled with topical medications, and there were no signs of disease progression. Repeated steroid administration subsequently resulted in a significant and refractory rise in IOP, which was deemed above target and placed these patients at high risk for either the development or progression of glaucoma.

Several studies to date have examined the frequency of steroid response after DEX-I administration and possible conservative and surgical treatment options. According to the MEAD study, steroid response occurred in 27.7% of patients treated with DEX-I 0.7 mg, and 41.5% needed pressure-lowering medication [16]. In a recent study, 26.2% of patients treated with DEX-I for DME or retinal vein occlusion showed an increase in IOP. All patients were adequately treated with IOP-lowering drops or iridotomy [27]. Callanan et al. reported an IOP increase in DME patients of ≥10 mmHg following DEX-I administration in 34.3%, and 39.2% of those needed IOP-lowering medication [28].

An extensive review of the literature has revealed that steroid response after DEX-I is a common side effect, though it is typically manageable with topical therapy. However, if surgical treatment is necessary, there is no consensus on which procedure might be deemed best for these patients.

Various surgical approaches have been reported in previous studies. In the MEAD study, 1.2% of patients treated with DEX-I required trabeculectomy or iridectomy, suggesting the presence of a narrow-angle component [16]. Sundhalkar et al. stated that 23.3% of patients treated with DEX-I had a steroid response, but only one patient required trabeculectomy to control IOP [29]. Callanan et al. reported that 0.6% of patients needed an additional IOP-lowering procedure, though the specific procedures performed were not disclosed [28]. Rajesh et al. reported that 0.5% of all patients treated with DEX-I required a filtering operation [15]. All of our patients had an open chamber angle upon gonioscopic examination, making iridectomy unlikely to have a significant IOP-lowering effect. While other surgical procedures are potential options, there are currently no definitive conclusions regarding the outcomes of glaucoma surgery in the studies mentioned above.

Studies on minimally invasive glaucoma surgery (MIGS) or laser surgery show moderate IOP reduction for patients with steroid response. Van Rijn et al. reported a 360° trabeculotomy reduced IOP from 30.8 mmHg to 11.2 mmHg after 1–2 years, with 98% achieving IOP ≤ 21 mmHg at 12 months, while ongoing steroid therapy did not impact the success of the procedure [30]. Zhou et al. reported a 54% failure rate for selective laser trabeculoplasty (SLT) over two years, with IOP decreasing from 27.6 mmHg to ~14.5 mmHg and a reduction in eye drops from 3.5 to 1.9 after 18 months [20]. Another study found that SLT lowered IOP by 45.4% with 1.5 pressure-lowering drops, while 89% of patients could continue DEX-I therapy [31]. Goniotomy or gonioscopy-assisted transluminal trabeculotomy (GATT) might be a further treatment option. Chen et al. reported 24-month results of 40 eyes after GATT surgery and 24 eyes after goniotomy in patients with steroid-responsive or uveitic glaucoma. GATT resulted in a mean IOP of 12.9 mmHg with 0.9 medications, while goniotomy achieved 14.3 mmHg with 1.8 medications. Both procedures provided moderate to good IOP reduction, although many patients still required medications [18].

These findings suggest that MIGS and SLT provide good short-term IOP reduction and may be suitable for moderate IOP increases. However, other surgical interventions may be required for significant, persistent elevations. The PFMS is considered to be a less-invasive glaucoma surgery—more invasive than MIGS but less so than traditional filtering glaucoma surgeries. Our study, which included eyes with PFMS implants, demonstrated superior outcomes in terms of success rates, despite the application of stricter success criteria. Additionally, the final IOP and medication use were lower compared with the results reported for the procedures discussed in the studies above [18,20,32].

As previously mentioned, most patients with a steroid response can achieve well-controlled IOP through medication, laser treatment, or MIGS. As a result, there are limited data on the use of major filtering surgeries or glaucoma drainage implants (GDIs) in this patient population. Studies have reported that filtering surgeries may become necessary for some patients after DEX-I treatment; however, they do not provide details on the outcomes of these glaucoma surgeries [19,33]. One available study specifically examines the outcome of non-penetrating deep sclerectomy (NPDS) after DEX-I. In a cohort of 60 young patients (mean age of 21.2 ± 8.2 years), NPDS was performed, which resulted in an IOP reduction from 34.2 ± 6.9 mmHg to 12.3 ± 2.6 at 12 months and to 13.6 ± 2.8 mmHg at 48 months. Complete success (IOP ≤ 21 mmHg without antiglaucoma medication) was 56.7%, and qualified success (≤21 mmHg with antiglaucoma medication) was 70% [34]. In comparison, our study showed better results for PFMS, with success rates of 87.5% (14/16) for IOP ≤ 21 mmHg with antiglaucoma medication and 75% (12/16) for IOP ≤ 21 mmHg without antiglaucoma medication. Mean pressure was 12.8 ± 2.1 mmHg after 12 months, and with a mean of 0.3 ± 0.8 IOP-lowering drops, our patients needed less medication 12 months after surgery. However, due to the young age of this group, comparability with our results is restricted. Although the study on the outcomes of NPDS in steroid responders demonstrated a good IOP-lowering effect and a reduced need for topical medication, it is important to consider that this procedure is more invasive. It should also be noted that NPDS can result in a larger filtering bleb, which may limit the ability to administer DEX-I repeatedly. In contrast, the PFMS creates smaller and more posterior filtering blebs, allowing for subsequent DEX-I treatments without complications. For this reason, we prefer to use the PFMS in patients with a persistent need for DEX-I and a steroid response, provided that a particularly low target IOP is not required. To the best of our knowledge, no other studies have specifically examined the outcomes of filtering surgeries for elevated IOP following intravitreal steroid treatment. Therefore, we are unable to compare our results with those of other studies.

Varying criteria should also be taken into account when comparing IOP values and success criteria in various studies. For this study, we chose criteria recommended by the World Glaucoma Association that included specific IOP thresholds and percentage IOP reductions (for A and B, ≥20%; for C, ≥25%; for D, ≥30% from baseline) [24]. In our case, the success rates did not change when the percentage reduction was taken into account. Since different studies use different IOP thresholds, percentage reductions, or a combination of both, discrepancies in the success rates can occur and thus make comparability difficult [35]. We also selected the success criteria as described to enable a comparison of our results with those from earlier studies on the PFMS. Bhayani et al. were able to report an average IOP reduction from 21.5 (19–28) mmHg to 13 (11–16) mmHg at 12 months after PFMS implantation for different glaucoma subtypes. The qualified and complete success rates (95% CI) after 12 months were, respectively, 74% (66–83%) and 58% (49–69%) for criterion A, 72% (63–82%) and 57% (48–68%) for B, 52% (43–63%) and 47% (38–58%) for C, and 29% (21–40%) and 26% (19–36%) for D [25,36]. In comparison, we report even better results for our specific patient cohort with steroid response at 12 months after implantation. One possible reason for this could be that our cohort included patients without manifest glaucoma damage, which may suggest a less aggressive form of glaucoma.

Another large study of 376 patients with mainly open-angle glaucoma reported complete success (which was defined as IOP between 6 and 17 mmHg without eye drops) in 74.8% of stand-alone non-refractory eyes and qualified success (6–17 mmHg with medications) in 92.4% of eyes [37]. The upper limit corresponds approximately to criterion B. Here, our complete and qualified success rates are slightly lower, at 68.75% and 81.25%, respectively. If the success rates for an IOP of <21 mmHg are considered, the complete and qualified success rates of 75.2% and 93.7% are almost identical to our results. Overall, it can be assumed that the success rates after PFMS implantation in patients with steroid response are comparable to the general success rates observed in broader patient populations, suggesting similarly effective outcomes for this specific cohort.

To date, no studies have specifically examined the outcome of PFMS following DEX-I administration. There is, however, one case report on the implantation of PFMS in a patient with ocular hypertension following DEX-I administration. XEN implantation, trabeculectomy, and laser trabeculoplasty were unsuccessful in this patient, but PFMS successfully lowered the IOP in the first 12 months after surgery, allowing for the continuation of DEX-I therapy [21]. In our cohort, 75% continued to receive DEX-I after PFMS implantation, with no impact on the success of the surgery or the number of postoperative IOP-lowering medications.

Various risk factors can increase the likelihood of a steroid response, such as a known history of glaucoma, which increases the risk of an earlier and more severe steroid response [38]. Other factors such as young patient age, previous retinal vein occlusion (RVO), known uveitis, and myopia with an axial length of more than 25 mm are associated with the development of SIOH [38]. Half of our patients had an underlying condition that may have contributed to the development of the steroid response, while RVO, diabetic retinopathy, and uveitis can also cause secondary glaucoma. In cases where patients had pre-existing glaucoma prior to DEX-I therapy, it was well controlled with medication. Nevertheless, this must be considered as a possible confounder. No clear correlation between the underlying condition and treatment failure was observed in our study; however, due to the small sample size, a more detailed analysis was not feasible.

Postoperative complications of other minimally or less-invasive procedures for treatment of steroid response did not differ significantly from those observed by us. Minor complications, such as ocular hyper- or hypotony and hyphema, were transient [18,20,30]. Bleb needling and revisions were rare and led to positive results in our case. Although one case required replacement of the PFMS due to an occluded intracameral tip, no further glaucoma surgery was required in all our patient cohort at the one-year mark. A recent study on the outcome of PFMS in general showed that if no new surgery is required within the first year, IOP is likely to remain well controlled even after three years [22,36].

When selecting a specific glaucoma surgical procedure, several factors must be considered. In addition to the severity of the glaucoma, ocular secondary diagnoses are key, as they can significantly influence outcomes. The patient’s overall health must also be taken into account. On the one hand, the procedure should effectively reduce IOP and the need for eye drops; on the other hand, postoperative risks and follow-up care should require minimal effort. Long surgery duration, general anesthesia, and frequent outpatient follow-up checks can be a significant burden, especially for patients with multiple comorbidities. Secondary diagnoses and necessary medications can also have an influence on the success of an IOP-lowering procedure. In the case of our cohort, this meant considering the potentially protective effects of dexamethasone on scarring and postoperative inflammation, and thus, failure of the operation. For these reasons, we consider PFMS implantation as a viable treatment option for steroid-induced rise in IOP. We recommend PFMS implantation for patients with elevated IOP following DEX-I administration, particularly when IOP-lowering therapy alone is insufficient or not tolerated. Compared with conventional glaucoma surgeries, PFMS creates smaller and more posterior filtering blebs, which allows for continued DEX-I treatments without complications. However, achieving IOP in the low teens can be challenging, so PFMS is not recommended when a particularly low target IOP is required. In our experience, PFMS provides stable IOP reduction with minimal postoperative complications, making it more effective than MIGS and safer than larger bleb-forming surgeries. Therefore, it represents a balanced option between these two approaches. In conclusion, we regard PFMS implantation as a viable treatment for steroid-induced IOP elevation, for the reasons outlined above.

There are several limitations to our study that must be considered. These include its retrospective design and non-comparative nature, which make it difficult to draw direct conclusions about how PFMS compares with other bleb-forming devices or filtering surgeries. To better assess the relative efficacy of PFMS, further randomized, comparative studies—particularly those involving MIGS—are needed to determine which procedure is most suitable for patients with a steroid response. Such studies would provide more solid evidence for treatment decisions in this patient cohort. Moreover, we can report only on a rather small group so far, as surgical treatment is not necessary for most steroid responders. This small sample size introduces a potential selection bias, as it is likely that only the more severe or refractory cases were chosen for surgery, limiting the ability to generalize the findings to the broader population of steroid responders. The relatively small cohort may also skew results, as more successful or less complicated cases may have been underrepresented, while those requiring surgical intervention represent a more challenging group of patients after DEX-I. Further, a distinction between SIOH and SIG was not made in the analysis due to the small cohort size, which limits our ability to assess the specific impact of each condition. In addition, certain variables, such as the endothelial cell counts, were not available, as these were not routinely recorded. It would be beneficial to identify various factors that influence the success or failure of the procedure. However, due to the small cohort size in our study, a sub-analysis of the demographic data and an evaluation of the potential impact of postoperative complications were not possible. Furthermore, we were unable to establish a correlation between the success or failure of the procedure and the underlying condition. Ultimately, we could report only on short-term results for now, and due to the limited follow-up period, it is not yet possible to comment on the long-term success rates or the sustainability of the outcomes over time.

## 5. Conclusions

In conclusion, PFMS surgery is a safe and effective procedure for patients with steroid response following DEX-I administration. It reduces IOP and prevents pressure spikes after repeated DEX-I treatments in most cases, allowing the continuation of therapy. Additionally, it minimizes the amount of pressure-lowering eye drops and is less invasive than traditional filtering surgeries, requiring fewer postoperative check-ups. If a low target pressure is required, PFMS might not be the most suitable device. Future research projects with a prospective design and a higher number of patients as well as longer follow-up are needed to extend the results reported here.

## Figures and Tables

**Table 1:** Demographics and clinical characteristics of patients undergoing PFMS implantation after IOP elevation due to DEX-I administration (BCVA = Best Corrected Visual Acuity; POAG = Primary Open-Angle Glaucoma; PEXG = Pseudoexfoliation Glaucoma).

Patient Characteristics	*n* = 16 (%)
Gender	
Male/Female	11 (68.8)/5 (31.2)
Age	
Mean (Min.–Max.)	70.63 (48–89)
Ethnicity—European descent	16 (100)
BCVA (logMAR)	
Mean (Min.–Max.)	0.51 (0–1.3)
Preoperative IOP	
Mean (Min.–Max.)	27 (16–38)
No. of IOP-lowering drops	
Median (IQR)	4.0 (3–4)
Acetazolamide	8 (50)
Pre-existing glaucoma diagnosis	
POAG/PEXG	3/1
SIOH/SIG	4 (25)/12 (75)
DEX-I-administration due to…	
Diabetic macular edema	6 (37.5)
Retinal vein occlusion with macular edema	5 (31.25)
Postoperative macular edema	3 (18.75)
Uveitis with macular edema	1 (6.25)
Macular edema of unknown cause	1 (6.25)
Further DEX-I administration after surgery	
Yes/No	12 (75)/4 (25)
Lens status	
Phakic	1 (6.25)
Pseudophakic	15 (93.75)
Previous VR surgery	8 (14.3)
Previous glaucoma surgery	4 (25)
Selective laser trabeculoplasty	1 (6.25)
Cyclophotocoagulation	3 (18.75)
Anaesthesia	
Local/general anaesthesia	16 (100)/0 (0)
Anticoagulation	4 (25)
Quadrant: superonasal	16 (100)

**Table 2:** Complications and following procedures related to PFMS implantation.

Complications Yes/No	5 (31.25%)/11 (68.75%)
Hypotony with choroidal detachment	1 (6.25%)
Needling with 5-fluorouracil administration	twice in one patient (6.25%)
Conjunctival revision due to encapsulated bleb	2 (12.5%)
PFMS exchange due to material deposition	1 (6.25%)

## Data Availability

All datasets generated during and/or analyzed during the current study are available from the corresponding author on reasonable request.

## Abbreviations

The following abbreviations are used in this manuscript:

PFMS	PreserFlo™ MicroShunt
IOP	Intraocular pressure
DEX-I	Intravitreal dexamethasone 0.7 mg implant
MMC	Mitomycin C
SIOH	Steroid-induced ocular hypertension
SIG	Steroid-induced glaucoma
BCVA	Best-corrected visual acuity
MIGS	Minimally invasive glaucoma surgery
SLT	Selective laser trabeculoplasty
GATT	Gonioscopy-assisted trabeculotomy
GDI	Glaucoma drainage implant
NDPS	Non-penetrating deep sclerectomy
RVO	Retinal vein occlusion
POAG	Primary open-angle glaucoma
PEXG	Pseudoexfoliation glaucoma
